# Gravity Wave Activity in the Stratosphere before the 2011 Tohoku Earthquake as the Mechanism of Lithosphere-atmosphere-ionosphere Coupling

**DOI:** 10.3390/e22010110

**Published:** 2020-01-16

**Authors:** Shih-Sian Yang, Masashi Hayakawa

**Affiliations:** 1 Independent researcher, Jhongli P.O. Box 9-11, Taoyuan 32099, Taiwan; 2 Hayakawa Institute of Seismo Electromagnetics, Co. Ltd. (Hi-SEM), University of Electro-Communications (UEC) Alliance Center, 1-1-1 Kojima-cho, Chofu, Tokyo 182-0026, Japan; hayakawa@hi-seismo-em.jp; 3 Advanced Wireless & Communications Research Center, UEC, 1-5-1 Chofugaoka, Chofu, Tokyo 182-8585, Japan; 4 Geoscent Technologies Inc., 2-8-11 Akasaka, Minato-ku, Tokyo 107-0052, Japan

**Keywords:** atmospheric gravity wave, the 2011 Tohoku earthquake, lithosphere-atmosphere-ionosphere coupling, reanalysis data, subionospheric VLF observations, stratopause temperature and height

## Abstract

The precursory atmospheric gravity wave (AGW) activity in the stratosphere has been investigated in our previous paper by studying an inland Kumamoto earthquake (EQ). We are interested in whether the same phenomenon occurs or not before another major EQ, especially an oceanic EQ. In this study, we have examined the stratospheric AGW activity before the oceanic 2011 Tohoku EQ (M_w_ 9.0), while using the temperature profiles that were retrieved from ERA5. The potential energy (E_P_) of AGW has enhanced from 3 to 7 March, 4–8 days before the EQ. The active region of the precursory AGW first appeared around the EQ epicenter, and then expanded omnidirectionally, but mainly toward the east, covering a wide area of 2500 km (in longitude) by 1500 km (in latitude). We also found the influence of the present AGW activity on some stratospheric parameters. The stratopause was heated and descended; the ozone concentration was also reduced and the zonal wind was reversed at the stratopause altitude before the EQ. These abnormalities of the stratospheric AGW and physical/chemical parameters are most significant on 5–6 March, which are found to be consistent in time and spatial distribution with the lower ionospheric perturbation, as detected by our VLF network observations. We have excluded the other probabilities by the processes of elimination and finally concluded that the abnormal phenomena observed in the present study are EQ precursors, although several potential sources can generate AGW activities and chemical variations in the stratosphere. The present paper shows that the abnormal stratospheric AGW activity has also been detected even before an oceanic EQ, and the AGW activity has obliquely propagated upward and further disturbed the lower ionosphere. This case study has provided further support to the AGW hypothesis of the lithosphere-atmosphere-ionosphere coupling process.

## 1. Introduction

It is recently agreed that precursory effects of an earthquake (EQ) could disturb the ionosphere (see recent books by [1,2,3,4]). Namely, anomalies in the very low frequency/low frequency (VLF/LF) propagation [5], formation of the sporadic E-layer [6], variation in the F-region plasma density, and total electron content (TEC) [7,8] have been observed and reported in the previous literature. The physical mechanism on how pre-EQ activities in the lithosphere can affect the ionosphere, i.e., lithosphere-atmosphere-ionosphere coupling (LAIC) process, is not well understood, but a few hypotheses have been proposed: (1) chemical (and associated electric field) channel, in which radon emanation from the lithosphere is the main player [9,10,11]; (2) atmospheric gravity wave (AGW)/acoustic wave (AW) channel that is excited by any perturbations with ground or atmospheric oscillations [12,13,14,15]; (3) electrostatic channel that is based on the generation of stress-activated positive holes that are near the ground surface [16,17] (stress changes affect several properties, including dielectric constants [18] and (re)orientation [19] of the electric dipoles that formed due to point defects [20,21]). A further introduction to these hypotheses can be found in [2,22]. A series of critical studies [23,24,25,26] has pointed out that the electric field/current, as proposed in the first chemical and the third electrostatic channel is weak, and that the penetration from the lithosphere into the ionosphere is negligible. Additionally, as compared with the first chemical and the third electrostatic channel, much observational evidence has been mainly accumulated in the subionospheric VLF/LF data to support the second AGW hypothesis [12], and thus we think that the AGW channel is the most promising candidate as the LAIC mechanism.

The precursory VLF/LF propagation anomalies usually appear a few days to about one week before an EQ [27,28]. We briefly list some important observational facts here: (1) shifts in terminator times [29]. The modulation in terminator times is enhanced by preexisting planetary waves with periods of 2–3, 5, and 10 days [30]; (2) reductions in nighttime amplitude of the VLF signal [5]; (3) enhancements of nighttime fluctuation [5], especially in the frequency range of AGW/AW in the VLF fluctuation spectra [27,31]; and, (4) Doppler shift in the VLF signals [32]. The authors of [29,33] have made a series of theoretical computations and demonstrated that those anomalies can be explained by a descent of a few km in the VLF reflection height (~85–90 km during quiet times) before an EQ. Furthermore, this descent is considered to result from AGWs [14] and, thus, precursory AGW activities modulate the VLF signals in various ways.

The papers that are mentioned above report the perturbations in the lower ionosphere, but unfortunately those observations are “indirect” (except the Doppler shift [32]) evidence of the AGW hypothesis, since no associated information in the regions of the lithosphere and atmosphere has been extensively studied. In consideration of this problem, several papers have attempted to find the link between seismo-ionospheric disturbances and lithospheric/atmospheric parameters. Some related phenomena have been concluded, such as the enhancements of AW (1–10 min.) and AGW (10–100 min.) components in the ground motions [34], short-term crustal movements [35] (which also accompany the generation of seismic electric signals [36,37]), the fluctuations in the AGW range in surface atmospheric pressure as well as ULF magnetic field [38,39], and anomalies in the mesospheric ozone density [40]. These papers provide certain evidence to the AGW hypothesis in the LAIC process.

However, we notice that the research in the stratosphere (middle atmosphere) is still absent in previous LAIC studies. In our preceding paper [41], the stratospheric AGW potential energy before the 2016 Kumamoto EQs (maximum moment magnitude of 7.2) in Japan has been evaluated from the ERA5 temperature profiles in the altitude range of 15–50 km. We have concluded that the stratospheric AGW activity is enhanced above the EQ epicenter during the week before the EQs. Besides, subionospheric perturbations with the use of our VLF network observation are observed during the same period [33]. Such good tempo-spatial coincidence between these two precursory anomalies in the stratosphere and lower ionosphere lends further support to the AGW hypothesis of the LAIC process. Even so, two questions need to be clarified here: (1) The Kumamoto EQ is a single event, for which we were successful in identifying the link between the stratospheric and ionospheric regions. Does this kind of stratospheric AGW anomaly take place before any other EQ? (2) The Kumamoto event is a series of inland EQs, and so how about the situation for an oceanic EQ? In this paper, we report the same analysis, as we have done in the Kumamoto event [41], to investigate the stratospheric AGW activity before the oceanic 2011 Tohoku EQ. Where convective weather systems and mountains can excite AGWs, we have some detailed discussions regarding the meteorological and topographic effects to eliminate those potential sources of wave activity. We will also proceed to a comparison of the stratospheric results with the subionospheric VLF observations, and we study the LAIC process while taking different precursors already reported into account in order to argument our conclusion.

## 2. EQ Treated in This Paper

A major EQ occurred over the offshore area of Tohoku, Japan (geographic coordinates: 38.10° N, 142.86° E, 24 km depth) at 05:46 UTC on 11 March 2011. This EQ was a reverse-fault type EQ with a moment magnitude (M_w_) of 9.0, and numerous studies of precursors for this EQ have been reported (see summary in [42]). A series of foreshocks were recorded in addition to the main event. The initial, and most powerful, foreshock (M_w_ 7.3) occurred at 38.33° N, 143.28° E, and 8 km depth at 02:45 UTC on 9 March 2011, about two days before the main shock.

## 3. Methodology

### 3.1. Gravity Wave and Its Potential Energy

We have designed a series of procedures to evaluate the AGW activity during the period prior to the EQ. Those details are found in our preceding paper [41], and we briefly repeat some key points here.

The influences of gravity wave on the atmosphere are manifested in both the temperature and wind field profiles. The total wave energy (E_0_) is composed of a summation of the kinetic (E_K_) and potential (E_P_) energies, which correspond to the fluctuations in wind fields and temperature, respectively [43]. The linearity between kinetic and potential energies [43,44] enables us to estimate the AGW activity by E_P_ alone from vertical temperature profiles, using

(1) $$E_{P}=\frac{1}{2}{\left(\frac{g}{N}\right)}^{2}\bar{{\left(\frac{T^{\prime }}{\bar{T}}\right)}^{2}}$$

where g is the gravitational acceleration constant and N is the Brunt–Väisälä frequency. A 2–10 km (the vertical wavelengths of stratospheric AGWs [45]) band-stop filter was used to filter out the wave components from the ERA5 temperature profiles T to get the background temperature $\bar{T}$. Subsequently, the perturbation term $T^{\prime }$ was calculated by subtracting $\bar{T}$ from T, i.e., $T^{\prime }=T-\bar{T}$. All of the variables, except g, are functions of altitude z and they are derived from the temperature profiles. The variance term $\bar{{\left(T^{\prime }/\bar{T}\right)}^{2}}$ is calculated within a layer of 2-km thickness, as

(2) $$\bar{{\left(\frac{T^{\prime }}{\bar{T}}\right)}^{2}}=\frac{1}{z^{max}-z^{min}}\int_{z^{min}}^{z^{max}}{\left(\frac{T^{\prime }}{\bar{T}}\right)}^{2}dz$$

where $z^{max}$ and $z^{min}$ are the top and bottom altitudes of each layer.

### 3.2. ERA5 Reanalysis Data and Construction of E_P_ Matrix

We have used the temperature profiles that were retrieved from ERA5 to calculate E_P_ as defined in Equation (1). The results will be further discussed with the ozone mass mixing ratio (concentration) and wind components that are also retrieved from ERA5 in this paper. ERA5 is an atmospheric reanalysis dataset produced by the European Centre for Medium-Range Weather Forecasts (ECMWF). This dataset assimilates model forecasts and real observations to provide an estimate of the true states of the atmosphere from the near surface to ~80 km altitude with 0.01 hPa. Further information regarding ERA5 can be found in ERA5 data documentation [46].

In this study, we have retrieved the ERA5 temperature profiles and constructed a four-dimensional E_P_ matrix for the following period and region: (1) date from 1 February to 31 March 2011; (2) latitude from 25° N to 50° N; (3) longitude from 120° E to 160° E; and, (4) altitude from 15 to 50 km. A second reference E_P_ matrix was constructed by averaging the values in March during 10 years of 2008–2018, except 2011. Although the temporal resolution is one hour, we have performed a moving average with 24-hour window to remove diurnal signals that we are not concerned with. The horizontal grid size is 0.3 degree in both longitude and latitude. On the other hand, the vertical resolution of ERA5 varies with altitude. We have performed a linear interpolation to obtain a fixed vertical grid size of 0.2 km.

### 3.3. Meteorological Analyses

Convective weather systems are known to excite gravity waves [47], and so meteorological effects should be identified, and we must carefully evaluate them before they can be excluded as potential sources. In our previous paper [41], we used the convective available potential energy (CAPE) that is derived from radiosonde data to identify the activity of convective systems around the EQ epicenter. The present case is an oceanic EQ, so radiosonde data are not available there, and thus we were obliged to use the CAPE values that were provided by ERA5.

The atmosphere is slightly unstable when the CAPE value is in a range from 500 to 1000 J/kg, and it is moderately (severely) unstable if the CAPE value exceeds 1000 (2500) J/kg. All of those conditions have indicated a certain occurrence of convective activities [48].

## 4. Observational Results on the Stratospheric Gravity Waves

### 4.1. Gravity Wave Activities above the EQ Epicenter

**Figure 1:**
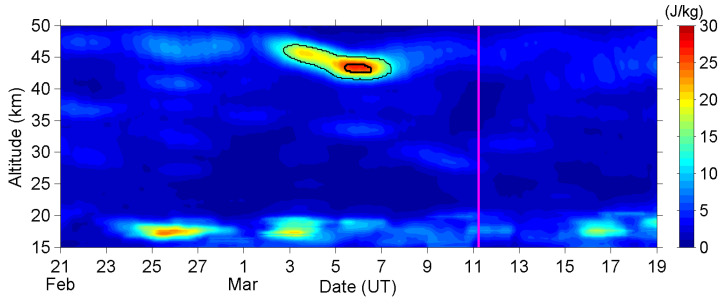
The time-altitude intensity of potential energy (E_P_). The magenta line indicates the occurrence time of the earthquake (EQ) (main shock). Thin/bold black lines circle the regions that the E_P_ values exceed the high-E_P_/extremely-high-E_P_ threshold (quadruple/octuple of the reference value), respectively.

Figure 1 shows the time-altitude intensity of E_P_ for the altitude from 15 km to 50 km above the EQ epicenter. The E_P_ values around the tropopause (about 16–18 km altitude) are, in general, larger than at other altitudes. The temperature inversion near the tropopause caused this enhanced band [49], and we are not concerned with it here. Our focus is on the regions, where E_P_ was enhanced several days before the EQ. For illustrating the significance of the E_P_-enhanced region, we circled the “high-E_P_” and “extremely-high-E_P_” regions in the figure, where the E_P_ values were larger than the quadruple and octuple of the reference value, respectively. These thresholds are different from the ones in our Kumamoto case [41], and we will discuss this difference later. As shown in Figure 1, E_P_ was significantly enhanced around the stratopause (about 43–47 km altitude) during 3–7 March, approximately 4–8 days before the EQ. This enhancement first appeared at 47 km altitude on 3 March and then descended to 43 km altitude while E_P_ reached its maximum value during 5–6 March. Afterward, E_P_ started to decay and the peak altitude of E_P_ returned to a higher level of 45 km. Thus, the wave activity was reduced a few days before the EQ day, and kept low during the week after the EQ.

### 4.2. E_P_ Maps

**Figure 2:**
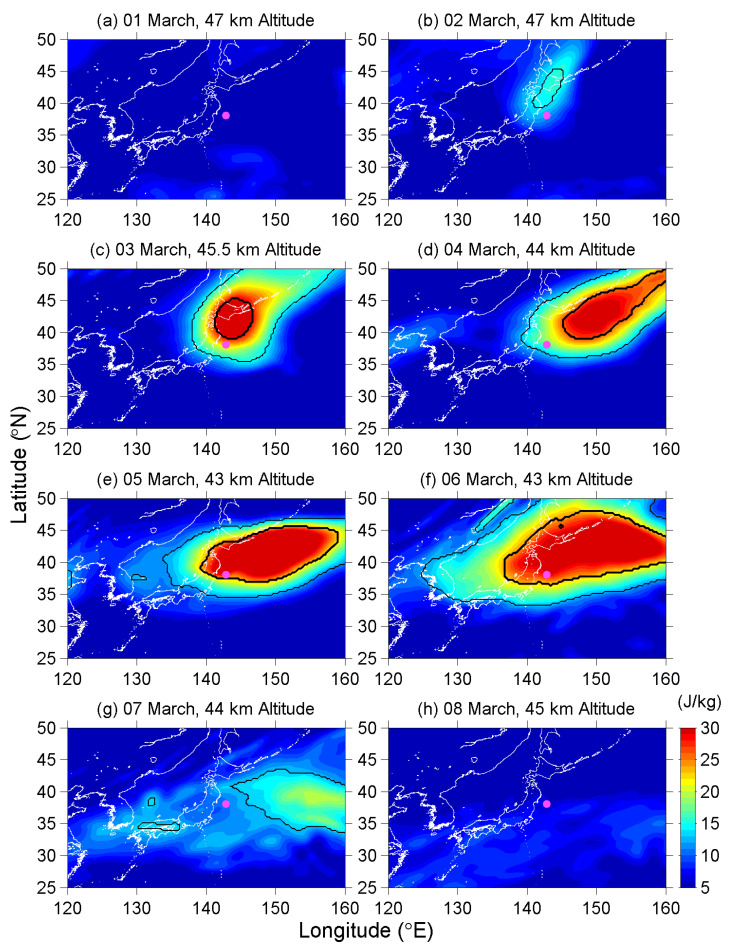
The E_P_ maps from 1 to 8 March. Thin/bold black lines circle the areas that the E_P_ values exceed the high-E_P_/extremely-high-E_P_ threshold, respectively. The location of the EQ epicenter is marked by a magenta dot.

We now turn to another view of the longitude-latitude intensity. Figure 2 shows the daily maps at the peak E_P_ altitude of from 1 to 8 March to illustrate the local spatial effect of this stratospheric response. Since the peak altitude varied between 43 and 47 km during this period as seen in Figure 1, the altitude of these maps in Figure 2 also changes with dates.

On 1 March in Figure 2a, the wave activity was low over northeast Asia and Pacific offing. Subsequently, an active region appeared around the Japanese Tohoku and Hokkaido area on 2 March (Figure 2b). The wave activity intensified quickly, and then exceeded the extremely-high-E_P_ threshold on 3 March (Figure 2c). This active region further expanded in all directions but mainly eastward on the following days (Figure 2d–f), and covered a wide area with the dimensions of more than 2500 km (in longitude) by 1500 km (in latitude) on 6 March. Being coincident with the result in Figure 1, the E_P_ values around the Tohoku area were sustained at a high level, although the highest value was located at the northeast of the EQ epicenter. After the most developed day on 6 March, the wave activity decayed on 7 March, and the E_P_ values that were around the EQ epicenter no longer exceeded the higher E_P_ threshold value (Figure 2g). Finally, the active region disappeared on 8 March (Figure 2h), and the wave activity remained in a calm state until one week after the EQ day (although the figures of the following days are not shown here).

### 4.3. Wavelength of the AGW Activities

The wavelength of the present AGW activity could be determined while using the temperature deviation ($T^{\prime }$) profiles (see an illustration in [41]). The $T^{\prime }$ profiles are somewhat similar to the E_P_ profiles, though those are not shown here. The vertical and horizontal wavelengths were approximately 6–7 km and 400–500 km, respectively, when the wave was most active during 5–6 March.

## 5. The Response of the Stratopause to the Precursory AGW Activity

Where the E_P_ value is mainly controlled by $T^{\prime }$ in Equation (1), the high-E_P_ region in Figure 1 represents a large temperature deviation around the stratopause. This implies that the stratopause is likely to be modified by the present AGW activity.

**Figure 3:**
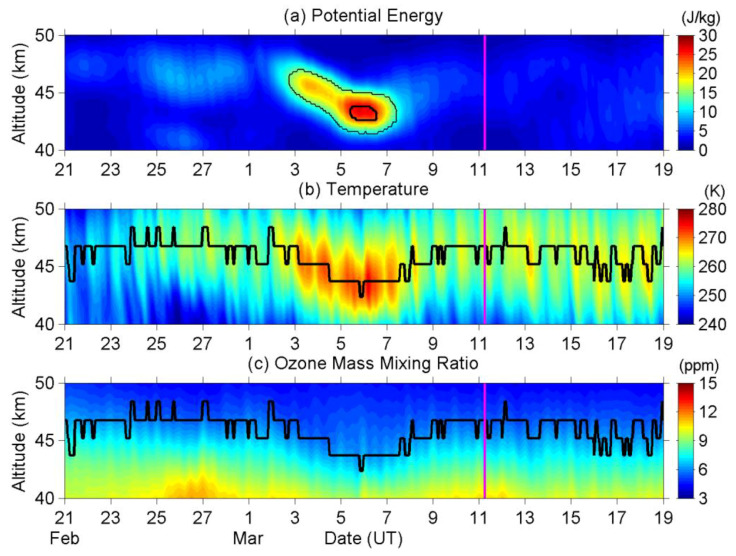
The compared plots of (**a**) E_P_ as shown in Figure 1, (**b**) temperature, and (**c**) ozone mass mixing ratio. The stratopause height is also displayed by a bold curve in both panels (**b**,**c**). The EQ occurrence is indicated by the vertical magenta line.

Figure 3a repeats the time-altitude intensity of E_P_ from 40 km to 50 km altitude, as shown in Figure 1. Figure 3b plots both the corresponding temperature profile and stratopause height. The stratopause height is defined as the altitude of the highest temperature between 40 and 50 km altitude in the present study. The curve of stratopause height is rough (not smooth), since the vertical resolution of the original ERA5 dataset is about 1.5 km around this altitude (though the resolution is much better in the lower atmosphere and we have made an interpolation with 0.2 km to estimate E_P_ values in the present study). The stratopause was mostly located at 46.8 km altitude until 2 March, and then it started to descend simultaneously with the enhancement of E_P_ on 3 March. The stratopause reached its lowest height at 43.8 km from late 4 March to early 7 March, and then gradually recovered to its original height at 46.8 km afterward. Besides, the stratopause temperature was enhanced from 260.33 K (mean value of 1–2 March) to 270.24 K (5–6 March) and then backed to 260.49 K (9–10 March) during this period. We have further checked the ensemble spread (uncertainty) for ERA5 temperature that is defined by its Ensemble of Data Assimilations (EDA) system, and the spread is about 0.7 K around the stratopause, which is much smaller than the evident heating of ~10 K. We can consider the heating here as a true anomaly that cannot be attributed to statistical error in the ERA5 reanalysis. In summary, the stratopause was lowered and also heated, while E_P_ was enhanced during the EQ-preparation period.

Ref. [40] have reported the depletion (enhancement) of ozone concentration in the lower (upper) mesosphere before the 2015 Gorkha Nepal EQ. We are also interested in the variation in the upper stratospheric ozone before the 2011 Tohoku EQ, since we have found the anomalies of stratopause in the present study. Figure 3c refers to the ozone mass mixing ratio from 40 to 50 km altitude that was retrieved from ERA5. The ozone mixing ratio at 45 km was reduced from 6.77 ppm (1–2 March) to 5.87 ppm (5–6 March) and then increased to 6.61 ppm (9–10 March), while the stratopause was heated during this period. The ensemble spread is about 0.04 ppm around the stratopause, which is much smaller than the magnitude of the anomaly. We further notice that, even beyond the EQ-preparation period, a negative correlation exists between the ozone mixing ratio and temperature around the altitude of 40–45 km. The possible mechanism and causal relationship of these variations on the physical and chemical parameters around the stratopause will be discussed in detail later.

## 6. Verification of the Variations at the Stratopause Using TIMED/SABER Data

Figure 3 has revealed the remarkable variations at the stratopause before a major EQ that have never been reported by previous literature. No further comparison or validation on the reliability of its stratospheric temperature and ozone data is available as of the moment this paper is finished given that the ERA5 is still a quite new dataset (~2 years). Here, we employ the temperature and ozone profiles that were observed by the SABER (Sounding of the Atmosphere using Broadband Emission Radiometry) instrument onboard the TIMED (Thermosphere, Ionosphere, Mesosphere Energetics, and Dynamics) satellite, to verify the results that were obtained using ERA5 data in the present study. The TIMED/SABER dataset has not been assimilated into ERA5, so it can impartially examine the reliability of ERA5. We learned that the TIMED/SABER dataset is a scattered (not gridded) dataset, the observational track passes through the longitudinal range of 120°–160° E (as shown in Figure 2) two to four times per day. Importantly, on the critical days of 5–6 March, the tracks passed overhead the Tohoku area. We have collected the data on each day within 120°–160° E and 25°–50° N, and then performed a two-dimensional (in both latitude and longitude) interpolation, to obtain the daily vertical temperature profiles above the EQ epicenter.

**Figure 4:**
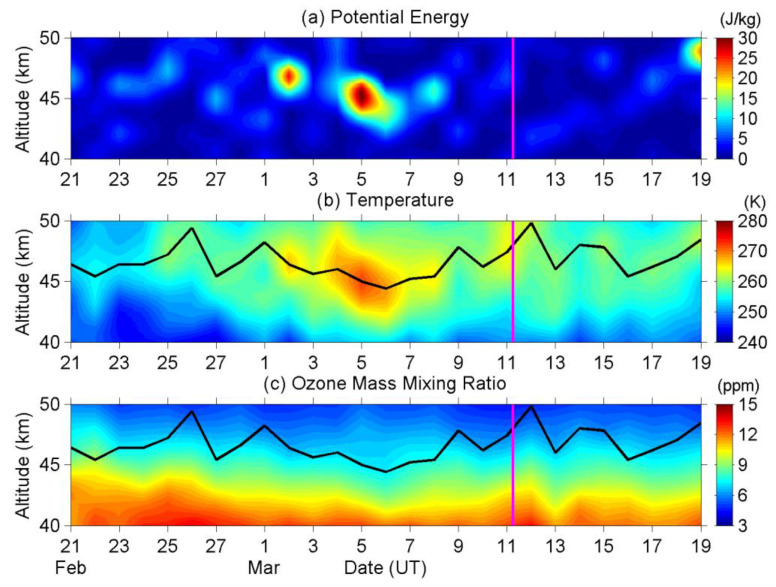
Same as Figure 3, but observed by TIMED/SABER.

Figure 4 shows the same parameters within the same range and period as Figure 3, but is observed by TIMED/SABER. The enhancement of AGW activity, the descent and heating of the stratopause, and the reduction of ozone are all evident. The stratopause height descended from 46.4 km (2 March) to 44.4 km (6 March) and then ascended to 46.2 km (10 March) altitude. The stratopause temperature was heated from 263.00 K (1–2 March) to 270.18 K (5–6 March), and then cooling to 260.29 K (9–10 March). The ozone mixing ratio at 45 km altitude was reduced from 7.98 ppm (1–2 March) to 7.24 ppm (5–6 March) and then increased to 7.92 ppm (9–10 March). The accuracies and precisions of SABER measurements at 45 km altitude are 1.0 K and <0.3 K for temperature, and 10% and 2% for ozone mixing ratio, respectively [50], with these being smaller than the magnitude of the observational anomalies.

In summary, both datasets show a descent of stratopause height of about 2–3 km, heating of stratopause temperature of about 10 K, and a reduction of ozone concentration of about 9–12%, although there is an obvious bias between the two ozone profiles. These TIMED/SABER results (Figure 4) have verified the prominent phenomena that we have found while using ERA5 (Figure 3).

We have an additional note here. The gridded ERA5 dataset provides the temperature and ozone profiles right overhead the EQ epicenter every hour. In contrast to that, the observational track of TIMED/SABER passes the Tohoku area about every four days with different local time during its every visit. The data that are provided by ERA5 are more detailed (in both time and space) than TIMED/SABER, and this is the reason why we choose ERA5 as our primary dataset in the present study, and the results using ERA5 seem reliable after we have compared both the temperature and ozone profiles in Figure 3 and Figure 4.

## 7. Lower Ionospheric Perturbation as Seen from the VLF Network Observation

The lower ionospheric perturbation in the form of changes in VLF amplitudes for this EQ has already been published with the use of a Japanese VLF/LF network that is composed of nearly 10 observing VLF stations [51,52,53], and we will repeat only the essential points. With this network we have been monitoring the lower ionospheric perturbation in the form of VLF/LF propagation anomalies. Our main emphasis is EQs taking place inland of Japan, so that our target transmitters are JJI in Miyazaki, Kyushu, and JJY in Fukushima. In addition, simultaneously, signals from three foreign transmitters, including NWC (Australia), NLK (Jim Creek, USA), and NPM (Hawaii), have been measured. The details of our VLF system are found in [51,52,53].

### 7.1. Temporal Evolution of Ionospheric Perturbation

**Figure 5:**
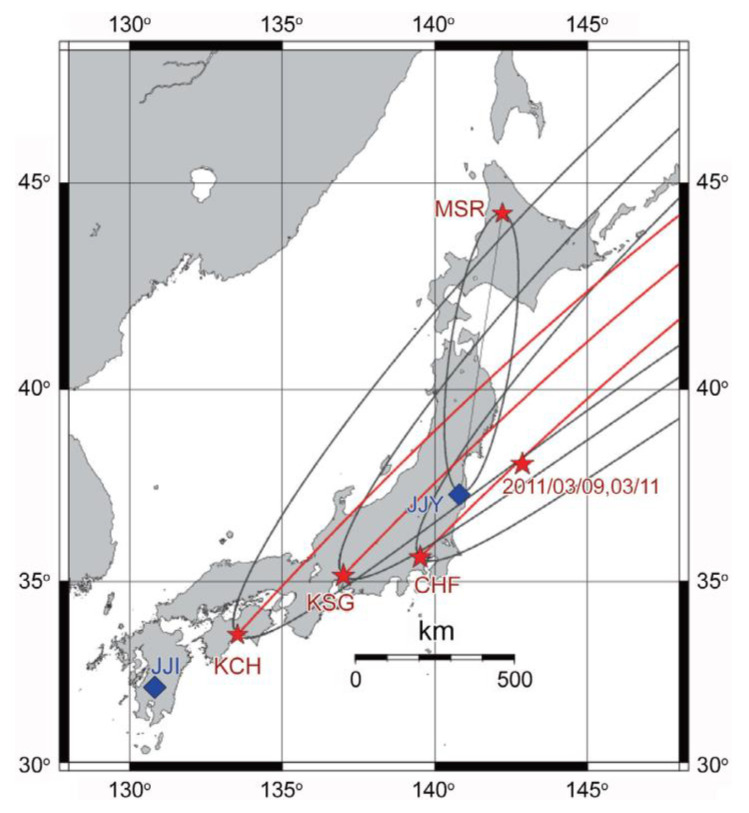
The relative locations of two Japanese very low frequency/low frequency (VLF/LF) transmitters (JJY (Fukushima) and JJI (Miyazaki) indicated by blue diamonds) and VLF receiving stations (Moshiri (MSR), Chofu (CHF), Kasugai (KSG), and Kochi (KCH) shown with red stars). Three red lines are the propagation paths associated with the American transmitter, NLK, together with their corresponding wave sensitive areas in thin black lines defined by Fresnel zones (elliptic zones). The most important one is the NLK-CHF path. For comparison, the great-circle path for JJY-MSR is also plotted. The epicenters of the main shock and its foreshock were nearly the same, as indicated by a red star in the sea. Taken from [52].

**Figure 6:**
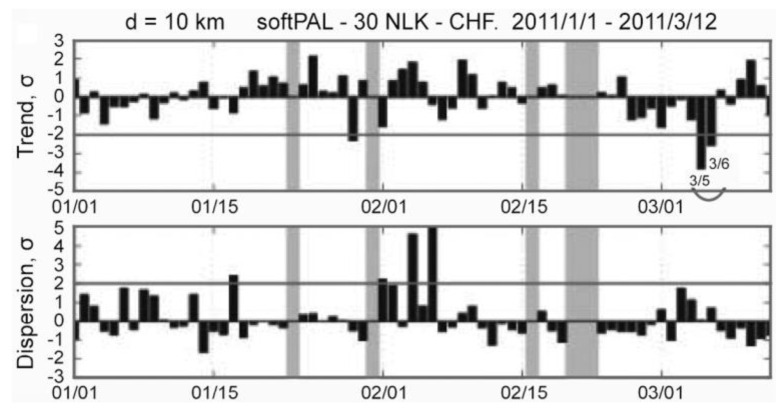
Temporal evolutions of the propagation characteristics for the NLK-CHF path. The top panel refers to the average nighttime amplitude (called trend), and the bottom, to the dispersion (in amplitude). All of these values are normalized by their corresponding standard deviation (σ). A clear anomaly as extraordinary depletion in trend is observed on 5 and 6 March. Taken from [52].

Figure 5 illustrates the propagation path from the American transmitter NLK to a few Japanese stations and their Fresnel zones. The most important path for this EQ is NLK-CHF (Chofu), which, as shown in Figure 5, is passing just over the EQ epicenter that is indicated by a red star. Figure 6 shows the corresponding propagation characteristics for the NLK-CHF path during a period of 1 January to 12 March 2011. The top panel illustrates the so-called “trend”, which is the nighttime average amplitude normalized by its running average preceding one month, and the second is dispersion that is defined by the fluctuation in amplitude. The details of these two quantities are found in [5]. A VLF anomaly is defined as depletion in trend (primary importance) and a nearly simultaneous enhancement in dispersion (secondary importance). It is seen from Figure 6 that there is a very remarkable depletion in trend (nearly −4σ) on 5 and 6 March before the EQ, which suggests that the lower ionosphere is very disturbed on these days. The corresponding responses have been detected at other stations of KCH (Kochi) and KSG (Kasugai), but with less extent, although not shown here.

### 7.2. Inferred Spatial Distribution of the Lower Ionospheric Perturbation

**Figure 7:**
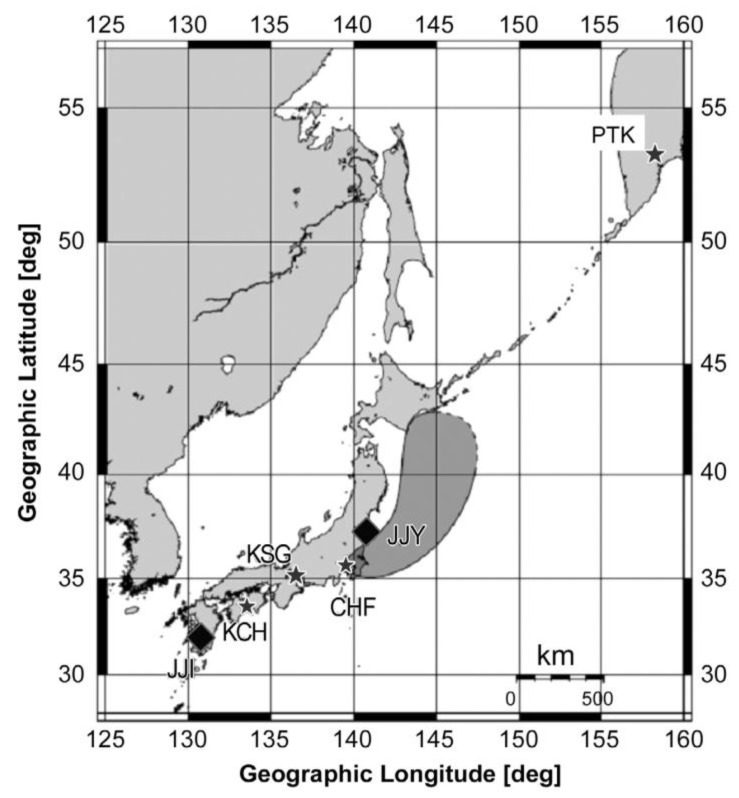
Illustration of the possible suggested region of the precursory seismo-ionospheric perturbation for the Tohoku EQ as inferred from a combination of propagation characteristics of all propagation paths we have studied. However, the north and east extension is quite uncertain due to the limitation of our VLF network. Taken from [52].

Based on the above VLF observational results, the authors of [52] have tried to infer the possible perturbed region of the lower ionosphere, and Figure 7 is their plot, while mentioning that the northern and eastern edges of the perturbed area (how much it is extended to the north and east) is quite uncertain because of the observational limitation of our VLF network. The information regarding the spatial distribution of the lower ionospheric perturbation is quite essential for us. We will examine the consistency between the stratospheric AGW activity and the lower ionospheric perturbation, and, further, make a comparison between the 2011 Tohoku EQ and the 2016 Kumamoto EQs in Section 8.

## 8. Discussion

In the present study, we have shown an enhancement of stratospheric gravity wave activity several days before the 2011 Tohoku EQ. Additionally, the stratopause has been modified during this period. We have to exclude other possibilities of the enhanced AGW activity before we conclude that the present one is a precursory AGW activity, as done in our previous paper [41]. Gravity waves could be triggered by weather systems and corrugated surfaces with sustained winds [47]. We will examine the meteorological effect while using quantitative and qualitative methods. On the other hand, the topographical effect (mountain waves) should be negligible in the current case, since the EQ epicenter and most of the active regions are both located in an oceanic area. Even so, we will still survey the mountain waves around Japan, especially in Tohoku. Besides, we have some comments on the physical and chemical variations around the stratopause. The possible mechanism of these variations and the causal relationship between different parameters will be discussed, and those discussions will be helpful in understanding what happened at the stratopause altitude. The stratospheric results will be compared with the subionospheric VLF observations, and we have introduced several precursors already reported to lend further support to the AGW hypothesis. Finally, we will also make a comparison of the precursory stratospheric AGWs between the 2011 Tohoku EQ and the 2016 Kumamoto EQs.

### 8.1. Elimination of the Meteorological and Topographical Effects

**Figure 8:**
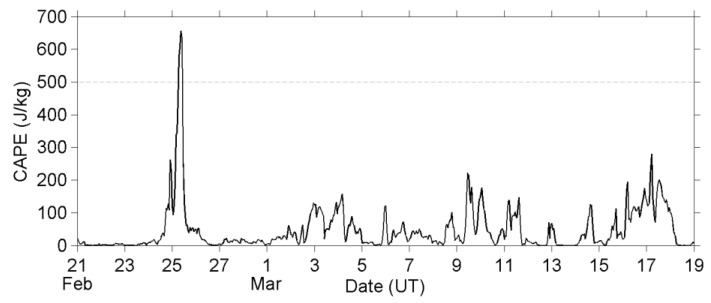
The convective available potential energy (CAPE) at the EQ epicenter.

Figure 8 plots the CAPE value at the EQ epicenter. The CAPE value has only exceeded 500 J/kg threshold on 25 February, and this threshold means that the atmosphere is slightly unstable with a low (but not zero) possibility of convective activity [48]. We further check the weather maps and reports that were issued by the Japan Meteorological Agency (JMA) to understand the meteorological situation, and find that a cold front passed the Tohoku area during the period of 24–25 February. Besides, thunderstorm activity was observed at Akita, while the cold front passed there, although the Akita weather station is about 300 km away from the EQ epicenter, and there is no on-the-spot weather report at the EQ epicenter, since this case is an oceanic EQ. We presume that convective systems have developed around the frontal region, and they passed through the EQ epicenter, and further resulted in a high CAPE value on 25 February, based on the above information. Besides, we notice that the weather was sometimes rainy or snowy in early and mid- March around the Tohoku area. Those precipitations were mainly stratiform, but not convective type, and the CAPE value at the EQ epicenter has ranged between 0 and 280 J/kg. No cold front had passed the EQ epicenter, and no thunderstorm had been reported around the Tohoku area from 26 February to 18 March.

Now, we return to the E_P_ values and look at Figure 1 more carefully. An unremarkable enhancement of E_P_ can be found around the tropopause and between 27 and 47 km altitude during the period of 25–27 February. This AGW activity is attributed to the convective systems that passed the EQ epicenter on 25 February, and it also faithfully demonstrates what (strength, duration, and spatial distribution) the meteorological AGWs excited by convective systems shall be over the Tohoku area in this season. Those meteorological AGWs have strength (in E_P_) of approximately double the background, which is much weaker than the anomalous AGW activity we have found on 3–7 March in Figure 1 and Figure 2. The AGW activities excited by those weather systems have a short lifetime, and the active regions always move speedily together with the corresponding weather systems, since mid-latitude systems develop and move very fast. However, the anomalous AGW activity on 3–7 March does not fit in with those properties of meteorological AGWs. Additionally, no convective activity was reported during this period in the Tohoku area, and further the CAPE value was low over the EQ epicenter. We can eliminate the possibility of the meteorological effect here.

Next, we discuss the topographical effect. The Ou Mountains are the main mountain range, being located in the Tohoku area. It has a length of ~500 km (NNE-SSW alignment), but its width is only ~35 km. Mount Iwate (39.85° N 141.00° E) with an elevation of 2038 m is the highest summit in the Ou Mountains. This mountain range is a good exciter for topographical gravity waves, because of its high and narrow ridges. However, a continuous wind flow perpendicular to the mountain range is needed to produce oscillations on the leeward side of the mountains and further excite mountain waves [47]. In March, the upper-air (means ~2000 m altitude here, which is close to the elevation of the Mount Iwate) wind in the Tohoku area is commonly westerly, which is almost perpendicular to the alignment of the maintain range. Orographic AGWs are therefore more likely to exist on the eastern side of the Ou Mountains. We can distinguish between topographical and meteorological generations of AGWs since mountain waves exhibit stationary patterns in contrast to the propagating waves produced by weather systems. Here, we can image the contribution from the topographical effect is a direct term because of its invariability, and the meteorological effect is an alternating term, since the weather is always time-varied. The AGW activity is thus a superposition of these two terms, even either or both of them is negligible. By taking the temporal average, the overall of the direct term and the mean of the alternating term are residuary. That is to say, the temporal average will be the superposition of the topographical effect (major contribution) and meteorological effect (minor contribution) under a moderate condition between stable and unstable atmosphere. Thus, the topographical effect is accentuated, and the temporal average of the AGW activity in March has already estimated as the reference E_P_ in Section 3.2.

**Figure 9:**
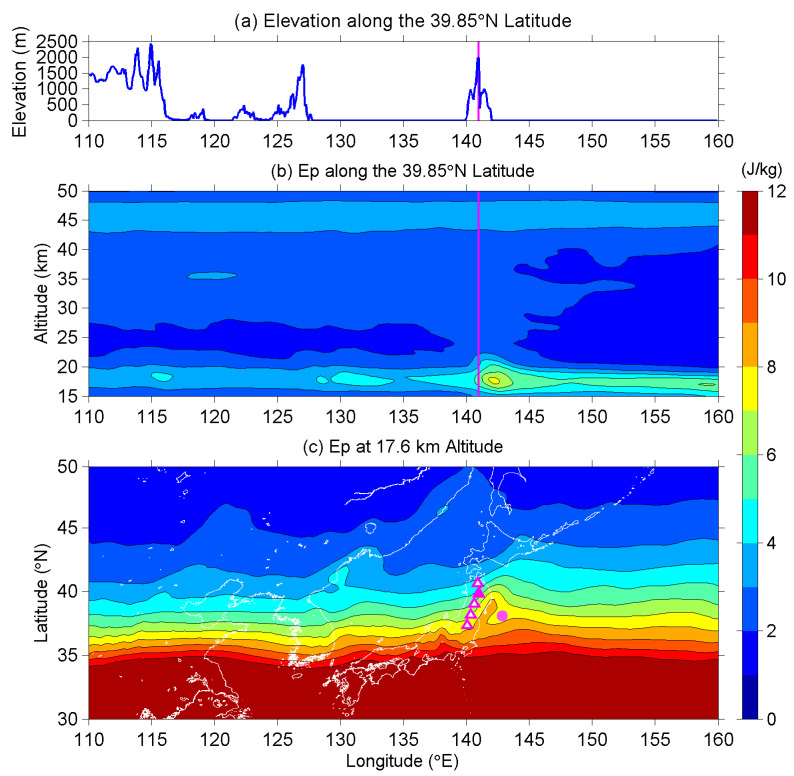
(**a**) The topographical elevation along 39.85° N, which is the latitude of Mount Iwate. The vertical magenta line further indicates the longitude (141° E) of Mount Iwate. (**b**) The longitude-altitude intensity of E_P_ along the 39.85°N latitude. The vertical magenta line again indicates the longitude of Mount Iwate. (**c**) The E_P_ map at 17.6 km altitude. Magenta triangles sketch the position of Ou Mountains, and the solid one is Mount Iwate. The magenta dot marks the EQ epicenter. The interval of contour lines is 1 J/kg in both (**b**,**c**).

We first look at the E_P_ on the longitude-altitude cross-section along 39.85° N, which is the latitude of Mount Iwate, to investigate the vertical and horizontal distributions of the mountain waves excited by the Ou Mountains. Figure 9a plots the ground surface elevation from 110° E to 160° E longitude, and Figure 9b is the corresponding longitude-altitude E_p_ intensity from 15 km to 50 km altitude along the 39.85° N latitudinal line. The most intense of AGW activity occurs at 142.2° E longitude and 17.6 km altitude in the lower stratosphere. The horizontal distance of this intense region is only 103 km from Mount Iwate, and it is considered to be the mountain waves excited by the Ou Mountains, since the wave activity is permanently located on the leeward side of the mountains. Additionally, from the literature (e.g., [47,54,55]), we learned that mountain waves are mainly distributed above the mountain ridges or within a range of few hundred km apart from the mountains. Besides, mountain waves usually have relatively low critical levels, and they break in the lower stratosphere, their influences on temperature and wind fields are confined below the lower stratosphere [56,57,58]. The intense wave activity that was observed on the leeward side of the Ou Mountains agrees with the properties of mountain waves on the basis of previous studies, and we can further confirm that the slight enhancements of E_P_ around 115°E and 130°E longitudes are also caused by mountain waves. We tried to plot the E_P_ map at this altitude in Figure 9c to realize the horizontal distribution of the waves at neighbor latitudes since we knew the mountain waves excited by the Ou Mountains are most active at 17.6 km altitude. The E_P_ values are quite large over low-latitudes because the plotted 17.6 km altitude is close to the tropopause there. The contour lines of E_P_ (with 1 J/kg interval) are mainly parallel to latitudinal lines, but are obviously curved around the Ou Mountains sketched by magenta triangles in the figure. E_P_ values enhance in the center of the mountains and the EQ epicenter, and this indicates mountain waves existing on the leeward (east-southeast) side of the Ou Mountains. One might also notice another active region (but not so significantly as the previous one) in the southwest direction of the Ou Mountains in Central Japan, which is caused by the high mountains (>3000 m elevation), but not related with the Ou Mountains.

Until now, we have fully understood the vertical and horizontal distributions of the mountain waves excited by the Ou Mountains, which are the main mountain range in the Tohoku area in Japan. The intense region of the mountain waves centers on 17.6 km in the lower stratosphere at 142.2° E longitude within a distance of 103 km from the expected source. The altitude and longitude of the topographical AGWs are both far from the anomalous AGW activity we have found in Figure 1 and Figure 2, with the latter one being located at 43–47 km altitude and 130–160° E longitude. We can also reject the possibility of the topography effect in the present study.

### 8.2. Comments on the Variations at the Stratopause

As seen in Figure 3c, the ozone concentration around the stratopause has reduced during the period of 2–9 March. The mechanism of this reduction in ozone should be discussed and determined to understand the relationship between the ozone reduction and the pre-EQ process.

A common solar-terrestrial phenomenon, energetic particle precipitation (EPP), is known to affect the ozone concentration in the upper atmosphere (e.g., [59,60]). Those energetic particles that precipitate into the atmosphere contribute to the important budgets of nitric oxides (NOx) and hydrogen oxides (OHx), and further catalyze the ozone loss in the Chapman cycle (ozone-oxygen cycle). This EPP effect on the atmospheric chemistry can mainly reduce the ozone concentration in the thermosphere and mesosphere but sometimes also in the upper stratosphere over high latitudes (see a review paper by [61]). Here we have to discuss the probability of EPP effect on the ozone depletion, as seen in Figure 3c. The geomagnetic latitude at the EQ epicenter is 30.93° N, and the apex height of the L-shell passing through the EQ epicenter is about 1.35 R_E_ (R_E_, the radius of the Earth). Particle precipitation has been reported over a low-latitude zone of L = 1.4 R_E_ for moderately disturbed geomagnetic conditions (30 nT <|Dst|<150 nT) [62]. There was a moderate geomagnetic storm on 1 March with a minimum Dst of −88 nT, which matches with the situation of possible EPP event at the EQ epicenter. However, we have no certain information and evidence for the occurrence of EPP event during early March 2011. Here, we leave the discussion on EPP effect temporarily and turn to another viewpoint of stratopause temperature.

From Figure 3b, we have found a significant enhancement of temperature at the stratopause before the EQ. During our calculation of E_P_ in Equation (1), this enhancement has remained in the temperature perturbation profile ($T^{\prime }$) after the filtering process and, thus, we obtained a large E_P_ value, as seen in Figure 3a. The E_P_ value has undoubtedly corresponded to the temperature perturbation. Even so, we have a concern about this temperature enhancement.

Photochemical processes (absorption of solar energy by ozone), but not dynamical processes (that include the gravity wave heating), mainly contribute the heating (energy budget) in the upper stratosphere [63]. Once the stratopause is heated by the increase of ozone, the enhanced temperature can result in a temperature perturbation and also a large E_P_ value like the ones that we have observed in the present study, even if the heating is not related with AGWs. This concern can be solved by checking the ozone mixing ratio in Figure 3c. If ozone dominates the abnormal heating, the ozone concentration and temperature will both enhance simultaneously. However, ozone has actually reduced during the period of 2–9 March. The temporal evolution is coincident, but the variation is negatively correlated with the temperature enhancement. This is quite relevant information for us, and we can now return to the postponed discussion on EPP effect. The influence of EPP-induced ozone depletion is found to cool the stratospheric temperature [61,64], although those previous studies focus on the stratospheric climate over a mid-to high-latitude region. However, this cooling process is against the result (temperature enhancement) that we have found in the present study. In addition, the influence of EPP events on the ozone concentration and the temperature is a planetary-scale effect, not the regional effect, as we have found. The probability of EPP-induced ozone depletion should be excluded in the present study.

The negative correlation between ozone concentration and temperature, as seen in Figure 3, can be satisfactorily explained again by the reaction rate of the Chapman cycle that the ozone loss rate is increased, while the temperature is also increased [65], and it is normal that the ozone concentration and the temperature is negatively correlated in the upper stratosphere [66], while the external budget of ozone (e.g., EPP event) is not taken into account. In the present study, dynamical processes shall heat the stratopause, and then the ozone loss rate has been modified, which further causes a reduction in the ozone concentration.

We further found that the zonal (u) component of the wind field at the stratopause had changed from westerly (eastward) to easterly (westward) on 5–6 March (the figure is not shown here), while the temperature enhancement and wave activity are both most significant. Wave breaking [47] might occur during this period, and energy and momentum transferred by the waves, influencing the thermal structure and the mean flow (wind field) around the stratopause [63,67]. However, we have also observed the perturbations in the ionosphere during the same period, which seems that the waves were not fully dissipated. Anyhow, the detailed mechanism on this coupling is something that implicates the dynamic processes of AGWs (e.g., wave-mean flow interaction, secondary wave generation), which is out of the scope of this paper and will be our future work.

We sum up the possible mechanism of those variations here before closing the discussions of stratospheric parameters. Precursory perturbation(s) with ground and/or near-ground atmosphere triggers AGWs in the atmosphere [12]. These AGWs propagate upward into the stratosphere, disturb the stratospheric temperature profile, and result in an enhancement of E_P_ (Figure 1 and Figure 2). The waves are likely to break and release energy around the stratopause, heat the stratopause (Figure 3b and Figure 4b), further modify the loss rate of ozone of the Chapman cycle [65], and thus result in an ozone reduction at the stratopause (Figure 3c and Figure 4c). On the causal relationship of these variations, the precursory AGW is the cause, the enhancement of stratopause temperature is the result, and the reduction in ozone concentration is a sequela (after-effect).

### 8.3. Comparison of the Stratospheric AGW Activity and the Subionospheric Perturbations

The results in Section 4 indicate that the stratosphere seems to be seismogenically perturbed by AGWs, especially on the specific days of 5 and 6 March, and those are compared with the ionospheric perturbation, as detected by VLF method in Section 7. A comparison of Figure 1 and Figure 2 for the stratosphere with Figure 5 and Figure 6 for the ionosphere suggests such a good agreement between the two, not only in time (4–6 March), but also in spatial distribution. This signifies that the stratospheric AGW activity is highly likely to be the agent of the lower ionospheric perturbation as the direct evidence for the AGW hypothesis in the LAIC process.

### 8.4. Additional Precursors for the 2011 Tohoku EQ

This section is intended to argue for the AGW hypothesis in the LAIC process. The most serious problem for the AGW channel is how AGWs are excited prior to the EQ, and we introduce several precursors to explain the possible solutions of exciting precursory AGW activity. Apart from medium-term (with lead time of a few months to a few years) precursors that are mainly based on crustal movements [35,68,69,70], groundwater [71], DC geomagnetic field [72,73] (note that the recorded DC magnetic field changes have been shown to reflect the emission of seismic electric signals activities [74]), minimization of the entropy change ΔS of seismicity under time reversal [75], which marks the approach of the system to a critical state [76], etc., we focus on short-term precursors to help convince the readers on our AGW hypothesis and also explore any link of our precursory AGW activity to certain ground or near-the-ground disturbances.

We start with the results of the upper region, ionosphere. Our VLF results of the lower ionospheric perturbation presented in the previous section are strongly supported by an anomaly of ULF (f = 0.01–0.03 Hz) magnetic field depression that peaked on 6 March [77]. This ULF depression is a phenomenon of the depletion of horizontal field components of magnetospheric ULF waves, which is attributed to the lower ionospheric perturbation [78], being common in nature with the subionospheric VLF propagation anomaly, and which is known to be sensitive to the pre-EQ effect. Being roughly coincident with this behavior in the lower ionosphere, the GPS TEC results indicate the F-region perturbation during 5–8 March, with the maximum on 8 March [79,80,81].

Next, we go to the atmospheric phenomena. A typical pre-EQ phenomenon in the atmosphere is impulsive ELF (f = 1–10 Hz) radiation, which has been really detected on 6 March on the basis of an ELF network observation in middle Japan and whose azimuthal direction was directed towards the EQ epicenter [82]. The mechanism of this new ELF effect is poorly understood, but it might be reasonable for us to suppose a kind of discharge due to some anomalous electric field situations in the lower atmosphere. The study by [80] observed OLR (outgoing longwave radiation) from satellite observations and found an enhancement in OLR on 7–10 March, with the highest activity on 8 March. Surface latent heat flux (SLHF) has also been measured from the satellite [81], which has shown several anomalous regions on 2–4 March, a little different from the dates of OLR [80].

Lastly, we consider the lithosphere. In [83], they analyzed the ULF lithospheric radiation with the data that were observed at Kakioka by a sophisticated critical natural time analysis. They have found that ULF radiation seems to be generated on 3 and 5–7 March. Another critical analysis that is based on the method of critical fluctuation has been applied to the same Kakioka data [84] and they have obtained nearly the same results as [83], supporting the generation of ULF radiation as a signature of pre-EQ fracturing process in the lithosphere. In agreement with these, Ref. [35], while using the GPS observation, have found that the change of longitudinal ground-surface movement exhibited a sharp increase over 6–10 March, and stayed flat on 3–6 March, in readiness to rebound.

In agreement with our promising AGW hypothesis of LAIC (Section 1), Ref. [85] have reported the two-day oscillations in nighttime NmF2 (maximum electron density of the ionospheric F2 layer) during 6–11 March observed at several ionosonde stations of Khabarovsk, Beijing, Wakkanai, and Kokubunji. They have further explained the mechanism of the two-day oscillations by modifications of the ionospheric dynamo electric field, which are caused by the influences of AGW breaking on the middle atmospheric wind fields, and the acting waves originate from ground motions before the EQ. In the present study, we have confirmed the existence of the precursory AGW activity in the middle atmosphere before the EQ. Our finding not only evidence the AGW assumption by [85], but also complements one lacking piece in the LAIC studies of the 2011 Tohoku EQ.

We have to comment on the effect of geomagnetic activity here, because there was a moderate geomagnetic storm on 1 March with Dst = −88 nT. The period of 3–6 March is just during its recovery phase, and the geomagnetic activity was rather quiet (with |Dst|< 30 nT). Hence, we think that this storm effect is negligible on 3–6 March and the good coincidence between the stratospheric and ionospheric perturbations may exclude this storm effect, as well because neutral particles are the main player in the stratosphere without any effect of plasmas, such as geomagnetic storms. The critical analyses of the ULF geomagnetic field cited above enabled us to study the criticality taking place in the lithosphere and we have found that criticality was really occurring during 3–8 March due to the pre-EQ fracturing process. Hence, different kinds of precursors that are observed during the period of 3–6 March are decidedly seismogenic. Even there was a series of foreshocks starting from 9 March before the main shock, the above anomalies were observed before the foreshocks, and they are pre-seismic (precursory), but not co-seismic phenomena; the latter kind is observed after an EQ occurs.

### 8.5. Comparison of the Precursory Stratospheric AGW Activity between the 2011 Tohoku EQ and the 2016 Kumamoto EQs

Through the investigation in the present study, we have found the second case where the stratospheric AGW activity has enhanced before a major EQ, following our first case in [41]. Here, we compare these two cases, to sum up some similarities and dissimilarities between them. Table 1 lists some parameters of the two EQ events and the properties of the precursory AGW activities. The information of subionospheric perturbations is listed as well for reference.

First of all, as we have wondered in Section 1, does the precursory AGW anomaly that we have found before the 2016 Kumamoto EQs also occur before another EQ, especially an oceanic EQ? The answer is yes. We have found the similar AGW anomaly before the oceanic 2011 Tohoku EQ in the present study. The precursory AGW is active 4–8 days before the Tohoku EQ, while that is 0–6 days before the Kumamoto EQs. However, the wave activities are most developed around 5–6 days before the main shock in both cases. The vertical wavelengths of the precursory AGWs are not quite the same; those are 6–7 km in the Tohoku case and 7–10 km in Kumamoto case, respectively. By contrast, the horizontal wavelengths are approximately 300–400 km and 400–500 km, which are approximately the same in these two cases. Besides, the precursory AGW activity first appears around the EQ epicenter and then expands eastward in both cases.

Another important fact is about the significance of the precursory AGW. The wave activity is more significant in the Tohoku case than the Kumamoto case. Although the background values of E_P_ are different between these two cases, because the EQ epicenters are located at different places and the EQs occur in different months. We have “normalized” the E_P_ values while using their reference values, and found that the highest E_P_ value during the most developed period is larger than octuple of the reference in the Tohoku case, but that is quadruple in the Kumamoto case. The difference in the significance might be caused by the dissimilarity of EQ magnitude (9.0 vs. 7.2), and/or the AGW dynamic processes as mentioned in Section 8.2. Especially, the possible wave breaking of the precursory AGWs is likely to explain that the stratospheric AGWs are more active before the Tohoku case than the Kumamoto case, but the subionospheric perturbations are more evident before the Kumamoto case [33] than the Tohoku case (see Section 7 and also Ref. [51,52,53]). The detailed mechanism of the different significances in the stratosphere and lower ionosphere has to be answered when we will study more cases in the future.

We also notice that the 2011 Tohoku EQ is a reverse-fault type EQ, and this is different from the 2016 Kumamoto EQs, which are a series of strike-slip type EQs. The occurrence of precursory phenomena between the EQs with different kinds of seismic focal mechanisms (reverse faults, normal faults, or strike-slip faults) is another interesting topic in the LAIC study, since some of the specific processes in the proposed LAIC hypotheses are never tenable in at least one kind of focal mechanism (e.g., the positive-pole theory in the electrostatic channel with a normal-fault type EQ). The difference in the focal mechanism between the 2011 Tohoku EQ and the 2016 Kumamoto EQs is quite essential for us. This is evidence that our theory has been applied to reverse-fault and strike-slip-fault EQs; so, the investigation of a normal-fault type EQ is necessary in the future.

## 9. Conclusions

In our previous papers, we have reported the abnormal stratospheric AGW activity [41] and also the subionospheric perturbations [33] before the 2016 Kumamoto EQs. The comparison of the tempo-spatial evolutions between these two precursory phenomena has further indicated that they are simultaneous and concentric anomalies, which support the AGW hypothesis of the LAIC process, although the stratospheric paper [41] is just a case study. We tried to perform the same analyses to learn whether the stratospheric anomaly exists or not in another seismic event, especially an oceanic EQ, as we have just done in the Kumamoto paper, on the basis of the ERA5 reanalysis data to compute the potential energy E_P_, to study the stratospheric AGW activity before the oceanic 2011 Tohoku EQ.

Before the Tohoku EQ, the AGW activity was significantly enhanced around the stratopause from 3 to 7 March (4–8 days before the EQ). Especially on 5–6 March (5–6 days before the EQ), the precursory AGW was the most active, and the highest E_P_ value exceeded the octuple of the reference value that was constructed by a 10-year’s mean. The present AGW activity is not correlated with any meteorological or topographical origin, and we have concluded that the AGW activity is a seismogenic effect. The active region first appeared around the Japanese Tohoku and Hokkaido area on 2 March, and then it expanded in all directions, but mainly toward the east on the following days of 3–7 March. Although there were a series of foreshocks before the main shock starting from 9 March, the abnormal AGW activity has occurred before the foreshocks. Therefore, the origin of the AGW activity is undeniably a pre-seismic, but not co-seismic, effect.

In contrast to the Kumamoto case [41], we have further found some physical and chemical variations at the stratopause in the present Tohoku case. The stratopause height was descended, the temperature was enhanced, the ozone concentration was reduced, and even the zonal wind direction was changed, all around the 43–47 km altitude, while the AGW activity was enhanced there during the period of 3–7 March. The precursory AGW activity explained those variations at the stratopause. The waves modify the temperature profile and thermal structure at the stratopause, and further result in a reduction in ozone concentration (see the detail discussions in Section 8.2).

Besides, the tempo-spatial evolution of the stratospheric AGW is found to be very consistent with the subionospheric perturbations. Both anomalies are most significant on 5–6 March, and are mainly distributed eastward of the EQ epicenter. It is highly likely that the lithosphere and lower atmosphere was really perturbed as a possible agent of AGW excitation by introducing the reported precursors in the atmosphere and lithosphere in Section 8.4, but it is needless to say that further study is required.

We have evidenced the simultaneous and concentric anomalies in the stratosphere and lower ionosphere that support the AGW hypothesis of the LAIC process through the two studied cases of the 2016 Kumamoto EQs and the 2011 Tohoku EQ, even though the seismic focal mechanisms are different in these two cases. We have also coordinated some similar and dissimilar properties between these two cases in Section 8.5, and the physical mechanisms that how Nature controls those properties are still unknown. However, those are just the beginning of the investigation on the precursory AGW in the stratosphere. More cases have to be studied to find out the rules of such an anomaly in the future.

## Figures and Tables

**Table 1:** A comparison of the EQ and stratospheric atmospheric gravity wave (AGW) parameters as well as the subionospheric information between the 2011 Tohoku EQ and the 2016 Kumamoto EQs. The EQ parameters are provided by JMA, and the subionospheric information is taken from [33,52].

	Tohoku (This Paper)	Kumamoto [41]
*(EQ parameters)*		
Date (D-day) & time of the main shock	0546Z 11 March 2011	1625Z 15 April 2016
Magnitude (M_w_)	9.0	7.2
Epicenter	Oceanic	Inland
Focal mechanism	Reverse fault	Strike-slip faults
*(Stratospheric AGW parameters)*		
Active period	3–7 March 2011 (D-8 to D-4)	9–15 April 2016 (D-6 to D-day)
Most developed days	5–6 March 2011 (D-6 to D-5)	9–11 April 2016 (D-6 to D-4)
Significance	~8 * reference value	~4 * reference value
Vertical wavelength	~6–7 km	~7–10 km
Horizontal wavelength	~400–500 km	~300–400 km
Expansion of the active region	All directions, mainly eastward	Eastward
*(Subionospheric perturbations)*		
Most perturbed period	5–6 March 2011 (D-6 to D-5)	7–12 April 2016 (D-8 to D-3)
